# 11*β*-HSD1 Modulates the Set Point of Brown Adipose Tissue Response to Glucocorticoids in Male Mice

**DOI:** 10.1210/en.2016-1722

**Published:** 2017-03-27

**Authors:** Craig L. Doig, Rachel S. Fletcher, Stuart A. Morgan, Emma L. McCabe, Dean P. Larner, Jeremy W. Tomlinson, Paul M. Stewart, Andrew Philp, Gareth G. Lavery

**Affiliations:** 1 Institute of Metabolism and Systems Research, University of Birmingham, Birmingham B15 2TT, United Kingdom; 2 Centre for Endocrinology, Diabetes and Metabolism, Birmingham Health Partners, Birmingham B15 2TH, United Kingdom; 3 Oxford Centre for Diabetes Endocrinology & Metabolism, University of Oxford, Churchill Hospital, Headington, Oxford OX3 7LE, United Kingdom; 4 Faculty of Medicine and Health, University of Leeds, Leeds LS2 9NL, United Kingdom; 5 School of Sport Exercise and Rehabilitation Sciences, University of Birmingham, Edgbaston, Birmingham B15 2TT, United Kingdom

## Abstract

Glucocorticoids (GCs) are potent regulators of energy metabolism. Chronic GC exposure suppresses brown adipose tissue (BAT) thermogenic capacity in mice, with evidence for a similar effect in humans. Intracellular GC levels are regulated by 11*β*-hydroxysteroid dehydrogenase type 1 (11*β*-HSD1) activity, which can amplify circulating GC concentrations. Therefore, 11*β*-HSD1 could modulate the impact of GCs on BAT function. This study investigated how 11*β*-HSD1 regulates the molecular architecture of BAT in the context of GC excess and aging. Circulating GC excess was induced in 11*β*-HSD1 knockout (KO) and wild-type mice by supplementing drinking water with 100 μg/mL corticosterone, and the effects on molecular markers of BAT function and mitochondrial activity were assessed. Brown adipocyte primary cultures were used to examine cell autonomous consequences of 11*β*-HSD1 deficiency. Molecular markers of BAT function were also examined in aged 11*β*-HSD1 KO mice to model lifetime GC exposure. BAT 11*β*-HSD1 expression and activity were elevated in response to GC excess and with aging. 11*β*-HSD1 KO BAT resisted the suppression of uncoupling protein 1 (UCP1) and mitochondrial respiratory chain subunit proteins normally imposed by GC excess. Furthermore, brown adipocytes from 11*β*-HSD1 KO mice had elevated basal mitochondrial function and were able to resist GC-mediated repression of activity. BAT from aged 11*β*-HSD1 KO mice showed elevated UCP1 protein and mitochondrial content, and a favorable profile of BAT function. These data reveal a novel mechanism in which increased 11*β*-HSD1 expression, in the context of GC excess and aging, impairs the molecular and metabolic function of BAT.

## Teaser

Using BAT from 11*β*-HSD1 KO mice treated with chronic GCs, our data show, in the context of GC excess and aging, 11*β*-HSD1 negatively impacts BAT functionality.

Brown adipose tissue (BAT) is adapted to expend chemical energy in the form of heat, predominantly through the action of the uncoupling protein 1 (UCP1), and has important roles in whole-body energy homeostasis (1, 2). Humans are recognized to have discrete depots of BAT that have diminished thermogenic potential in the context of chronic metabolic diseases of aging (3). Therefore, exploiting BAT function to enhance energy expenditure may improve metabolic profiles in individuals with metabolic diseases (4). BAT is activated by cold exposure and sympathetic tone, increasing the metabolic activity required for thermogenesis, with many hormonal factors playing a coordinating role.

Glucocorticoids (GCs) are powerful regulators of energy metabolism and recent data have revealed species differences between humans and mice. For humans, acute GC exposure increases, rather than suppresses, cold-induced BAT glucose uptake and thermogenesis via UCP1 (5, 6). However, chronic GC exposure, in excess of normal circadian rhythm, is considered to inhibit BAT function in both (7, 8). GC excess can repress the expression of genes essential to BAT function, including UCP1 (9–15), driving lipid accumulation and inhibiting sympathetic stimulation and cyclic adenosine monophosphate-dependent signaling to induce thermogenesis (16–18). Conversely, GC depletion after adrenalectomy or GC receptor antagonism stimulates BAT thermogenesis and weight loss in obese models via improved BAT functionality (7, 19, 20). Importantly, chronic GC excess in humans (*i.e.*, Cushing’s syndrome) causes metabolic disease with presentation including obesity and type 2 diabetes mellitus, with the consequences for BAT biology being largely unexplored (21).

The hypothalamic-pituitary-adrenal (HPA) axis determines circulating GC levels, with intracellular tissue GC levels further regulated by the activity of the enzyme 11*β*-hydroxysteroid dehydrogenase type 1 (11*β*-HSD1), which converts inactive 11-dehydrocorticosterone to active corticosterone in rodents (cortisone to cortisol in humans). Consequently, 11*β*-HSD1 expression can influence intracellular GC availability independently of circulating levels. 11*β*-HSD1 knockout (KO) mice are largely protected from the tissue-specific responses to circulating GC excess and demonstrate a critical role of the enzyme in transducing extracellular GC concentrations to intracellular signaling (22, 23). 11*β*-HSD1 is expressed in brown adipocytes and, when overexpressed *in vitro*, induces GC-mediated BAT dysfunction (24), 11*β*-HSD1 knockdown *in vitro* or after pharmacological inhibition *in vivo* enhances BAT function in the face of a high-fat diet challenge (24), and 11*β*-HSD1 KO mice have been shown to have an elevated core body temperature, again suggesting an influence on BAT function (25, 26).

Here, we examine the impact of GC excess and aging on the molecular architecture and mitochondrial activity of murine BAT, with the hypothesis that 11*β*-HSD1 plays an important role in determining the set point of GC sensitivity. We show that 11*β*-HSD1 upregulation, in response to chronic GC excess and aging, increases brown adipocyte GC exposure to impair BAT function. 11*β*-HSD1 KO mice resist GC-mediated suppression of UCP1 protein, BAT thermogenic gene-expression programs, and have preserved mitochondrial function and activity. Thus, we reveal a physiologically relevant mechanism of 11*β*-HSD1–mediated GC generation inhibitory to BAT function, which may have implications for our understanding of metabolic dysregulation in GC excess and aging.

## Materials and Methods

### Animal care, mouse strain, storage, and aging

Male mice (C57/BL6J) were group housed at 22°C for 10 (young) or 100 (aged) weeks. All mice were maintained in a standard temperature- (22°C) and humidity-controlled environment with a 12:12-hour light-dark cycle. Mice had nesting material and *ad libitum* access to standard animal chow and water. Corticosterone (CORT)- or 11-dehydrocorticosterone (11-DHC)-supplemented drinking water (100 μg/mL, 0.66% ethanol as a vehicle) was administered for 5 weeks. Mice were killed by cervical dislocation, tissue was collected, individual tissue weights recorded, and samples snap frozen in liquid nitrogen. Collections were all performed at 9:00 am. Experiments were conducted consistent with current UK Home Office regulations in accordance with the UK Animals (Scientific Procedures) Act 1986, and approved by the Animal Welfare and Ethical Review Body.

### Primary cell culture of brown adipocytes

Interscapular BAT was extracted and manually digested before placement in collagenase, and then incubated in a 37°C water bath for 40 minutes with 10 seconds of vortex every 5 minutes. After incubation, samples were vortexed and spun for 10 minutes at 1000 rpm and the supernatant discarded. The remaining pellet was resuspended in 1 mL of proliferation media [Dulbecco’s modified Eagle medium (DMEM)/F12 culture medium supplemented with 10% fetal calf serum and 1% penicillin-streptomycin] and aliquots plated. The cells were incubated at 37°C in 5% CO_2_ for 24 hours. The next day, the proliferation medium was removed and cells were washed with 1 mL of fresh proliferation medium before replenishment of 1 mL of proliferation medium to each well. Cells were incubated at 37°C in 5% CO_2_ for 48 hours, with medium replacement every 24 hours. After 48 hours, the proliferation medium was replaced with 1 mL of differentiation medium (DMEM/F12 culture medium supplemented with 1 nM triiodothyronine, 2 μM rosiglitazone, 166 nM human insulin, and 1 μM dexamethasone). The cells were allowed to differentiate at 37°C, 5% CO_2_, for 9 days more with the differentiation medium changed daily.

### Cell treatments

Differentiated brown adipocytes were treated in DMEM/F12 serum-free media, with CORT (1 μM; catalog no. 27840; Sigma) or ethanol as a vehicle control for 24 hours. Treatments with CL-316,243 hydrate (1 μM; catalog no. C5976; Sigma) were conducted for 5 hours with double-distilled water as a vehicle control.

### RNA extraction and quantitative reverse transcription polymerase chain reaction

Total RNA was extracted from adipose tissue, using TRI reagent (Invitrogen). RNA quality was determined by visualization on a 1.5% agarose gel and quantity was measured by nanodrop absorbance at 260 nm. Reverse transcription was conducted using 500 ng of RNA that was incubated with 250 μM random hexamers, 5.5 mM MgCl_2_, 500 μM deoxynucleotide triphosphates, 20 units of RNase inhibitor, 63 units of multiscribe reverse transcription, and ×1 reaction buffer. Reverse transcription was performed on a thermocycler set for the following conditions: 25°C for 10 minutes and 48°C for 30 minutes before the reaction was terminated by heating to 98°C for 5 minutes. Complementary DNA levels were determined using an ABI7500 system (Applied Biosystems); reactions were conducted in a 96-well plate in singleplex format. Primers and probes were purchased as Assay on Demand (FAM) products (Applied Biosystems). Total reaction volumes used were 10 μL containing Taqman Universal polymerase chain reaction (PCR) mix (Applied Biosystems). All reactions were normalized to 18s ribosomal RNA (VIC probe; Applied Biosystems). The real-time PCR (RT-PCR) was performed at the following conditions: 95°C for 10 minutes, then 40 cycles at 95°C for 15 seconds, and at 60°C for 1 minute. Data were collected as threshold cycle (Ct) values and used to obtain the change in Ct values.

### Histology of BAT

Freshly excised interscapular brown adipose depots were dissected, processed, and embedded in paraffin wax, from which 5-μm sections are cut for analyses via histological staining. Hematoxylin and eosin stains were applied to the sections. All images pertaining to histological analyses were viewed via light microscopy and photomicrographs were taken with a Leica imaging system using ×20 magnification.

## 11*β*-HSD1 enzyme assays in BAT explants

Mouse BAT was excised and tissue samples were incubated with 100 nm of 11-DHC diluted into 1 mL of serum-free media in glass tubes. Tracer amounts of [H^3^]11DHC (synthesized in house) were added and incubated for 4 hours (27). Upon cessation of the incubation period, steroids were extracted from the medium with dichloromethane, separated by thin-layer chromatography using chloroform and ethanol in a 92:8 ratio. The fraction of converted steroids was measured by scanning analysis using a Bioscan 2000 radioimaging detector. Conversion of 11-DHC to CORT was calculated and normalized to tissue weight.

### Mitochondrial copy number

DNA was isolated using a phenol/chloroform isomyl alcohol mix, precipitated, and pellet rehydrated into nuclease-free water. Samples were then measured for nucleic acid concentration using a Nanodrop spectrophotometer (Thermo Scientific) and diluted to normalized concentrations and volume (*i.e.*, 100 ng/μL). The NovaQUANT Mouse Mitochondrial to Nuclear DNA Ratio Kit (catalog no. 72621; Merck) was used to quantify mitochondrial DNA (mtDNA) as a ratio to nuclear DNA, per manufacturer instructions. This compares the levels of nuclear DNA with mtDNA in a sample using RT-PCR primers directed to both nuclear DNA and mtDNA of equivalent amplification efficiency, allowing expression to be compared as a ratio of nuclear DNA to mtDNA.

### Western immunoblotting

Protein lysates were collected in radioimmunoprecipitation assay buffer (50 mmol/L Tris, pH 7.4, 1% NP40, 0.25% sodium deoxycholate, 150 mmol/L NaCl, 1 mmol/L EDTA), 1 mmol/L phenylmethylsulfonyl fluoride, and protease inhibitor cocktail (Roche, Lewes, United Kingdom) stored at −80°C for 30 minutes, defrosted on ice, and centrifuged at 4°C for 10 minutes at 12,000 rpm. The supernatant was recovered and total protein concentration was assessed by Bio-Rad assay (Bio-Rad Laboratories). Total proteins (25 μg) were resolved on a 12% sodium dodecyl sulfate-polyacrylamide gel electrophoresis (SDS-PAGE) gel and transferred onto a nitrocellulose membrane. Primary antibodies [UCP1: Research Resource Identifier (RRID) AB_2213764, catalog no. ab10983, Abcam; Mitoprofile OXPHOS Cocktail: RRID AB_2629281, catalog no. ab110413, Abcam; cyclic-AMP–related response element (CREB): RRID AB_331277, catalog no. 9197, Cell Signaling Technology; Phos-CREB Ser-133: RRID AB_256044, catalog no. 9198, Cell Signaling Technology; mouse anti-*β*-actin: RRID AB_306371, catalog no. A-5441, Sigma Aldrich; and rabbit anti-11*β*-HSD1: RRID AB_731458, catalog no. ab39364, Abcam]. Anti-mouse and anti-rabbit secondary antibodies (Dako) conjugated with horseradish peroxidase added at a dilution of 1:10,000. Equal loading of protein content was verified using *β*-actin and bands were visualized using the ECL detection system (GE Healthcare, United Kingdom). Autoradiograph films were scanned and bands were measured using ImageJ densitometry (https://imagej.nih.gov/ij/) and normalized to those of loading control (*β*-actin).

### Metabolic flux analysis by Seahorse XF

Primary BAT preadipocytes were isolated from culled mice, seeded at equal densities, and differentiated for 8 to 9 days. Respiratory output and mitochondrial stress tests were performed with BAT in supplemented XF-Assay media (25 mM glucose and 0.5 mM sodium pyruvate) at pH 7.4 and maintained for 1 hour at 37°C in 0% CO_2_ before Seahorse XF analysis (Agilent Technologies). Wells were normalized to protein concentration and expressed as picomole per minute per microgram protein. Mitochondrial function was assessed using the Seahorse XF Cell Mito Stress Kit (catalog no. 103015-100; Agilent Technologies), oligomycin (2.5 μM), carbonyl cyanide-4-(trifluoromethoxy)phenylhydrazone (5 μM), rotenone, and antimycin A (1.0 μM). Parameters were assessed as dictated by Seahorse Bioscience (Agilent Technologies): basal respiration = oxygen consumption rate (OCR) − nonmitochondrial respiration rate; maximal respiration = OCR with carbonyl cyanide-4-(trifluoromethoxy)phenylhydrazone − nonmitochondrial respiration rate; and adenosine triphosphate (ATP) production = OCR − OCR with oligomycin.

### Statistical analysis

Student *t* test or analysis of variance statistical comparisons were used with Prism version 5 (GraphPad Software). Data are presented as mean ± standard error of the mean. Two-way analysis of variance using the Bonferroni multiple comparison *post hoc* test compared treatments and genotypes together. Unpaired *t* test compared treatments or genotypes. Statistical analysis derived from RT-PCR data were determined using change in Ct values throughout.

## Results

### 11*β*-HSD1KO mice resisted GC-excess–mediated suppression of BAT

11*β*-HSD1 has emerged as a determinant of how GC excess manifests tissue-specific effects in liver, muscle, and white adipose tissue (28). We wanted to extend these data to examine novel aspects of *in vivo* BAT molecular biology with respect to the role of 11*β*-HSD1. To model GC excess, we supplemented the drinking water of wild-type (WT) and 11*β*-HSD1KO mice with 100 μg/mL CORT or vehicle for 5 weeks, as previously described (28). In this model, serum GCs were comparable between WT and 11*β*-HSD1 KO mice, with both demonstrating adrenal atrophy as a result of feedback repression of the HPA axis (18, 28–30).

**Figure 1.**
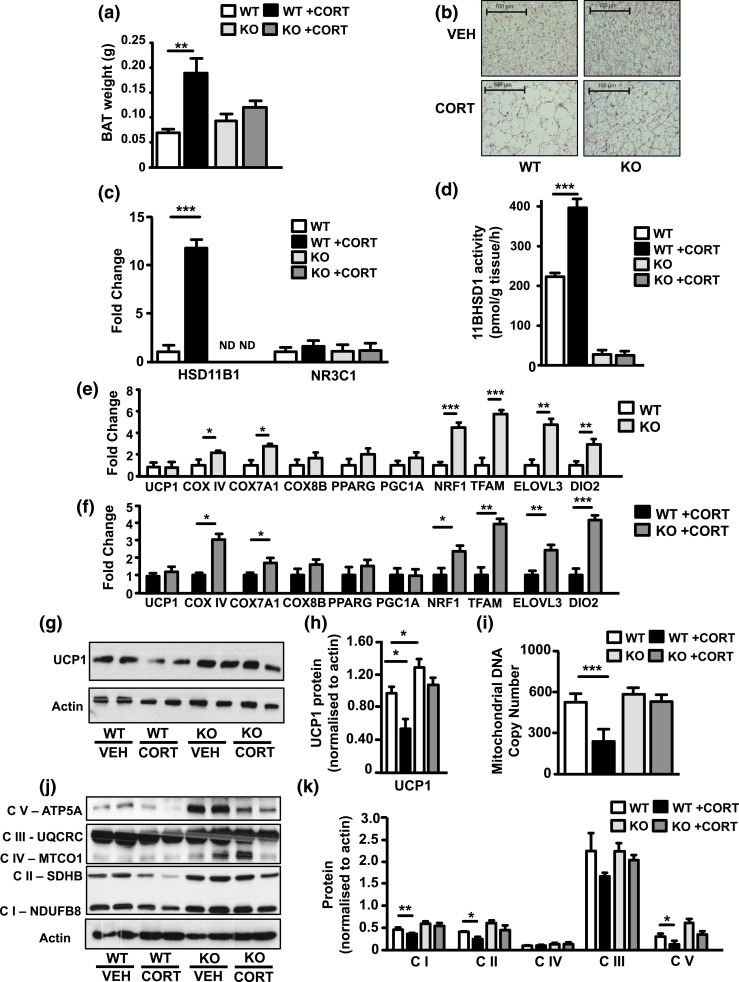
11*β*-HSD1 KO amplifies favorable stress response and mitochondrial metabolic activity of murine BAT. Ten-week-old WT and 11*β*-HSD1 KO mice were maintained for 5 weeks on vehicle or CORT-spiked drinking water. Upon cessation, interscapular BAT was excised and its weight recorded. (a, b) Hematoxylin-and-eosin staining was performed on formalin-fixed BAT. (c) RNA was isolated from the BAT and measured using quantitative reverse transcription PCR for 11*β*-HSD1 and GR (NR3C1). (d) 11*β*-HSD1 enzyme activity levels recorded in WT and 11*β*-HSD1 KO explants. (e, f) RNA from 11*β*-HSD1 KO and 11*β*-HSD1 KO plus CORT BAT measured for BAT regulators, components of mitochondrial respiration, and mitochondrial biogenesis, and compared with WT and WT plus CORT, respectively. (g, h) Protein expression of UCP1 measured by (g) SDS-PAGE and (h) quantified. (i) mtDNA copy number also was assessed in the extracted BAT. WT and 11*β*-HSD1 KO BAT protein observation of mitochondrial respiratory complexes with and without CORT were conducted by (j) SDS-PAGE and mitochondrial oxidative phosphorylation antibody cocktail. (k) Quantification results. Group sizes were 6 to 10 mice. All values are presented as mean ± standard error of the mean. (a, c, d, h, i, k) Assessed using two-way analysis of variance with Bonferroni *post hoc* test. (e, f) Analyzed using unpaired *t* test. *P < 0.05; **P < 0.01; ***P < 0.001.

Whereas the interscapular BAT depot weight of 11*β*-HSD1 KO mice was similar to that of WT mice, 11*β*-HSD1 KO BAT resisted archetypal lipid accumulation and the increase in BAT mass seen in GC-treated WT mice [Fig. 1(a)]. Hematoxylin and eosin staining showed that GC-treated WT BAT had increases in lipid droplet size compared with that of untreated WT and 11*β*-HSD1 KO mice. However, there was an increase in lipid droplet size in GC-treated 11*β*-HSD1 KO BAT compared with vehicle treated, but not to the same degree as in WT mice [Fig. 1(b)].

GCs can increase the expression and activity of 11*β*-HSD1 in WT mice and this study shows exposure of BAT to GC excess stimulates 11*β*-HSD1 transcription in WT mice but has no effect upon the GC receptor [GR (or NR3C1); Fig. (c)]. Increased BAT 11*β*-HSD1 expression was endorsed by showing increased levels of enzyme activity from WT BAT explants [Fig. 1(d)].

We next evaluated BAT status in untreated mice, using established expression profiles. UCP1 mRNA levels were unchanged compared with WT BAT, but 11*β*-HSD1 KO BAT did display a profile associated with enhanced BAT function, with increased mRNA abundance of genes integral to efficient metabolic function and mitochondrial biogenesis, such as ELOVL3, DIO2, TFAM, and NRF1 [Fig. 1(e)]. Because GCs repress *β*-adrenergic stimulation of BAT, UCP1 expression, and overall thermogenic capacity, we reasoned that deficiency of 11*β*-HSD1 would help preserve BAT function in the context of GC excess. Again, UCP1 mRNA did not show a discernible difference between 11*β*-HSD1 KO and WT BAT. However, as in untreated mice, GC-treated 11*β*-HSD1KO mice retained an elevated expression profile of markers seen in the untreated state [COX7A1, COXIV, TFAM, NRF1, ELOVL3, and DIO2; (Fig. 1(f)].

UCP1 mRNA levels do not always predict the level of protein; therefore, we assessed UCP1 protein and showed that although GC repressed UCP1 expression in WT BAT, 11*β*-HSD1KO BAT was resistant to this and retained elevated levels [Fig. 1(g) and 1(h)]. 11*β*-HSD1KO BAT was also protected from GC-induced decreases in mitochondrial copy number [Fig. 1(i)]. We extended this to evaluate subunits of the mitochondrial respiratory chain [Fig. 1(j) and 1(k)]. Although GC repressed WT BAT mitochondrial subunit expression, in line with a decreased mitochondrial copy number, 11*β*-HSD1KO had significantly preserved subunits of CI, CII, and CV, indicative of protection from the deleterious effects of excess GC on mitochondrial capacity. Thus, the absence of 11*β*-HSD1 was mildly protective in the basal state, and became more prominent with chronic GC excess.

Previous studies have demonstrated that 11*β*-HSD1KO mice are also protected from the effect of circulating 11-DHC excess in various tissues (28, 31). Here, we show that this effect is also evident for BAT, with 11*β*-HSD1 KO mice exposed to 11-DHC–supplemented drinking water having greater levels of UCP1 and expression markers indicative of retained mitochondrial and BAT function compared with 11-DHC–treated WT mice [Supplemental Fig. 1(A–D)].

### 11*β*-HSD1 regulates brown adipocyte sensitivity to GC

Having shown the impact of 11*β*-HSD1 deficiency on the molecular characteristics of BAT in WT and 11*β*-HSD1 KO mice, we explored the cell autonomous sensitivity of brown adipocytes to GCs and temperature. Using differentiated primary brown adipocyte cultures derived from 11*β*-HSD1 KO and WT mice, we examined responses of cells treated with 1 µm of CORT for 24 hours.

**Figure 2.**
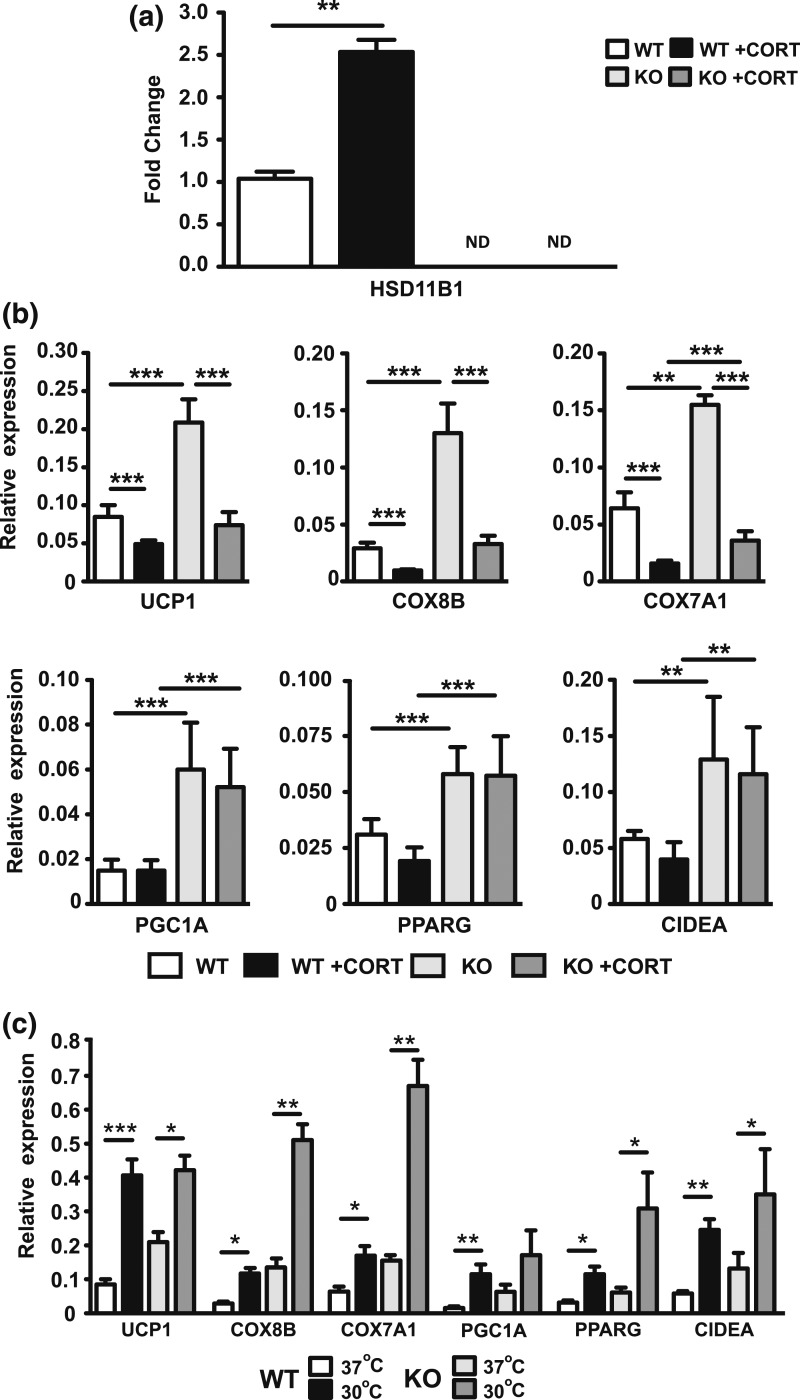
11*β*-HSD1 KO protection from GC exposure in brown adipocytes is cell autonomous. (a) RNA for 11*β*-HSD1 was measured using quantitative reverse transcription PCR in WT and 11HSD1 KO mice treated with or without CORT-spiked drinking water. Primary brown adipocytes from WT and 11*β*-HSD1 KO mice were cultured and differentiated. (b) RNA analysis of key BAT regulators and mitochondrial metabolic factors from BAT maintained at 37°C with and without CORT was measured by quantitative reverse transcription PCR. (c) Comparison of quantitative reverse transcription PCR data from WT and 11*β*-HSD1 KO brown adipocytes stored at 30°C and 37°C. Group sizes were six to eight mice. (a–c) Statistical assessment was performed using two-way analysis of variance with Bonferroni *post hoc* test. All values are presented as mean ± standard error of the mean. *P < 0.05; **P < 0.01; ***P < 0.001.

We confirmed *in vitro* and *in vivo* 11*β*-HSD1 mRNA induction in WT cells in response to GCs [Fig. 2(a)]. We extended our analysis to show, *in vivo*, that GCs have the effect of suppressing markers of brown adipocyte function [Fig. 2(b)], in agreement with previous studies (24). Brown adipocytes derived from 11*β*-HSD1 KO mice had augmented expression of BAT metabolic and mitochondrial markers, with increased UCP1, COX8B, COX7A1, PGC1A, PPARG, and CIDEA expression [Fig. 2(b)]. Although basal expression of BAT markers was elevated in 11*β*-HSD1 KO cells, GC treatments did suppress expression in both WT and KO mice for UCP1, COX8B, and COX7A1, whereas, PGC1A, PPARG, and CIDEA, in both WT and KO cells, did not respond to GC treatment [Fig. 2(b)].Cold induces the sympathetic nervous system and *β*-adrenergic signaling to stimulate the BAT thermogenic program (32). To explore further a cell autonomous role for 11*β*-HSD1 in modulating responses to cold via thermogenic gene programs, we exposed WT- and 11*β*-HSD1 KO-derived primary brown adipocyte cultures to a cooler culture temperature (30°C). WT BAT-derived adipocytes showed a significant induction of the thermogenic transcriptional program when cells were cultured at 30°C [Fig. 2(c)]. Similarly, 11*β*-HSD1 KO-derived adipocytes showed again that the thermogenic program was expressed to a greater degree than in WT cells [Fig. 2(c)].

**Figure 3.**
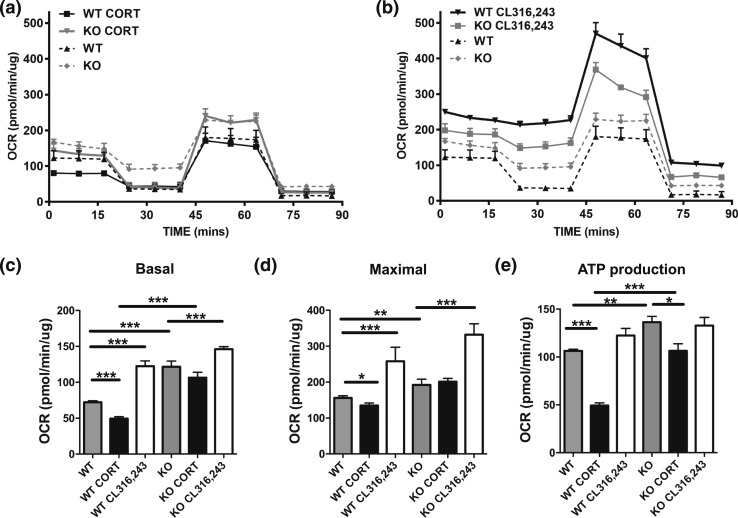
11*β*-HSD1 KO protection from GC exposure in brown adipocytes is cell autonomous. Primary brown adipocytes from WT and 11*β*-HSD1 KO mice were cultured and differentiated. (a, b) Basal OCR and mitochondrial assessment was measured (a) with and without CORT or (b) with and without CL316,243. (c–e) Respiration parameters were calculated for (c) basal, (d) maximal, and (e) ATP production conditions. Group sizes were three to five mice. All values are presented as mean ± standard error of the mean. (a–e) Statistical significance was calculated using two-way analysis of variance with Bonferroni *post hoc* test. *P < 0.05; **P < 0.01; ***P < 0.001.

We next examined how the expression signature related to a functional output as assessed by cellular oxygen consumption rate (OCR) as a measure of energy homeostasis in the presence of CORT and the *β*-3 adrenergic receptor agonist CL316,243 [Fig. 3(a) and 3(b)]. Untreated cells showed that 11*β*-HSD1 KO brown adipocytes had significantly increased basal, maximal, and ATP production levels compared with WT adipocytes, demonstrating an elevated level of respiration [Fig. 3(c)]. CORT excess significantly depressed the basal and maximal rates of WT but not 11*β*-HSD1 KO adipocytes. However, 11*β*-HSD1 KO adipocytes were impaired by CORT when measuring ATP production, suggesting the protective effect does not fully extended to coupled respiration, although it is still significantly higher than the CORT treated WT. CL316,243 was able to induce both WT and 11*β*-HSD1KO basal and maximal OCR in cells at proportional rates. Conversely, ATP production in WT and 11*β*-HSD1KO adipocytes treated with CL316,243 failed to reach significance in comparison with untreated cells [Fig. 3(c–e)].

### Elevated BAT 11*β*-HSD1 expression in aged mice

**Figure 4.**
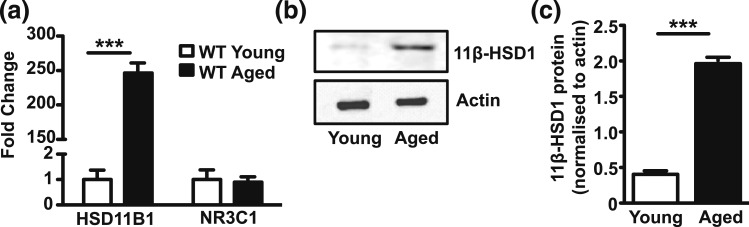
11*β*-HSD1 expression is increased in aged mice maintained at standard animal house conditions. Aged (100 weeks) WT and 11*β*-HSD1 KO mice were collected and BAT excised. (a) RNA levels of 11*β*-HSD1 and GR measured by quantitative reverse transcription PCR. (b, c) Protein levels of 11*β*-HSD1 were recorded along with *β*-actin as a loading control. Group sizes were 10 to 12 mice. All values are presented as mean ± standard error of the mean. (a–c) Statistical significance was calculated using unpaired *t* test. *P < 0.05; **P < 0.01; ***P < 0.001.

Given that 11*β*-HSD1-mediated GC metabolism plays a role in determining the molecular set point of BAT thermogenic and mitochondrial capacity, we reasoned that a lifelong loss of 11*β*-HSD1 (*i.e.* chronically attenuated GC signaling) would enhance molecular markers of BAT function. To test this, we examined archived BAT tissue from 100-week-old 11*β*-HSD1 KO mice that were fed a standard rodent chow under standard animal house conditions. First, we examined BAT 11*β*-HSD1 expression in aged WT mice compared with isogenic young mice and observed that both mRNA and protein were markedly elevated compared with young WT mice, without changes in the expression of the GC receptor [Fig. 4(a–c)].

### Elevated molecular markers of BAT thermogenic potential in aged 11*β*-HSD1 KO mice

**Figure 5.**
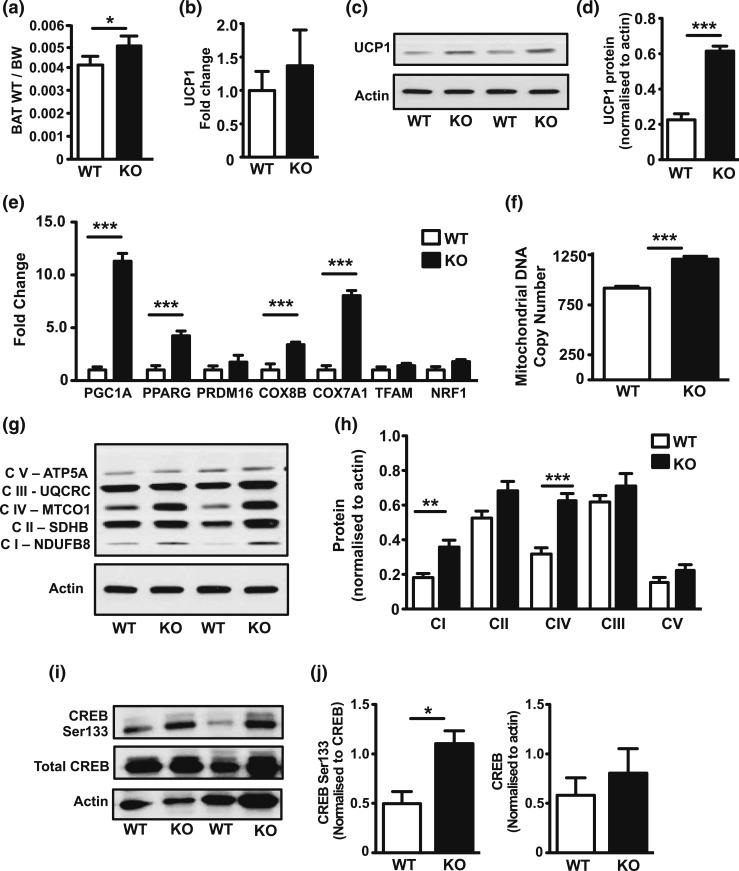
11*β*-HSD1 KO BAT shows enhanced molecular markers of mitochondrial function during aging. Aged (100 weeks) WT and 11*β*-HSD1 KO BAT was removed. (a) BAT weight recorded and expressed as a ratio of total body weight (BW). (b–d) Levels of UCP1 in both RNA and protein were measured in aged WT and 11*β*-HSD1 KO BAT. (e) RNA expression of stated integral components of BAT mitochondrial function. (f) mtDNA expressed as a ratio to nuclear DNA measured in BAT collected from WT and 11*β*-HSD1 KO aged mice. (g–j) SDS-PAGE of protein in aged WT and 11*β*-HSD1 KO BAT performed for oxidative phosphorylation subunits (g, h) and CREB phosphorylation (i, j). Group sizes were 8 to 12 mice. All values presented as mean ± standard error of the mean. (a–j) Statistical significance was calculated using an unpaired *t* test with significance at *P < 0.05, **P < 0.01, and ***P < 0.001.

Recent data show that elevated 11*β*-HSD1 expression in BAT impairs mitochondrial fatty acid oxidation capacity of BAT (33). As such, elevated 11*β*-HSD1 and increased intracellular GC availability in BAT from our aged mice may have acted to suppress maximal BAT potential. Aged 11*β*-HSD KO mice had increased interscapular BAT mass compared with aged WT mice [Fig. 5(a)]. Again, we found no significant difference in UCP1 mRNA levels but found threefold greater UCP1 protein levels compared with WT mice, suggesting increased potential for UCP1-mediated thermogenic activity [Fig. 5(b–d)]. In support of this, BAT from aged 11*β*-HSD1 KO mice also displayed increased expression levels of markers important to thermogenic potential and mitochondrial function [Fig. 5(e)]. We next examined mitochondrial copy number and protein content. Aged 11*β*-HSD1 KO BAT had an elevated mitochondrial copy number, endorsed by elevated levels of mitochondrial respiratory subunits, particularly CI and CIV [Fig. 5(f–h)].

Finally, we show elevated CREB serine-133 phosphorylation as evidence of increased functional potential of BAT from 11*β*-HSD1 KO mice, because it is demonstrated to play a role in SIRT3-mediated activation of PGC1A and increased oxygen consumption within brown adipocytes [Fig. 5(i) and 5(j)] (34). These data highlight, in the context of normal murine aging physiology, that 11*β*-HSD1 can act as a restraint to BAT through repression of genes controlling thermogenic capacity and mitochondrial turnover or biogenesis.

## Discussion

**Figure 6.**
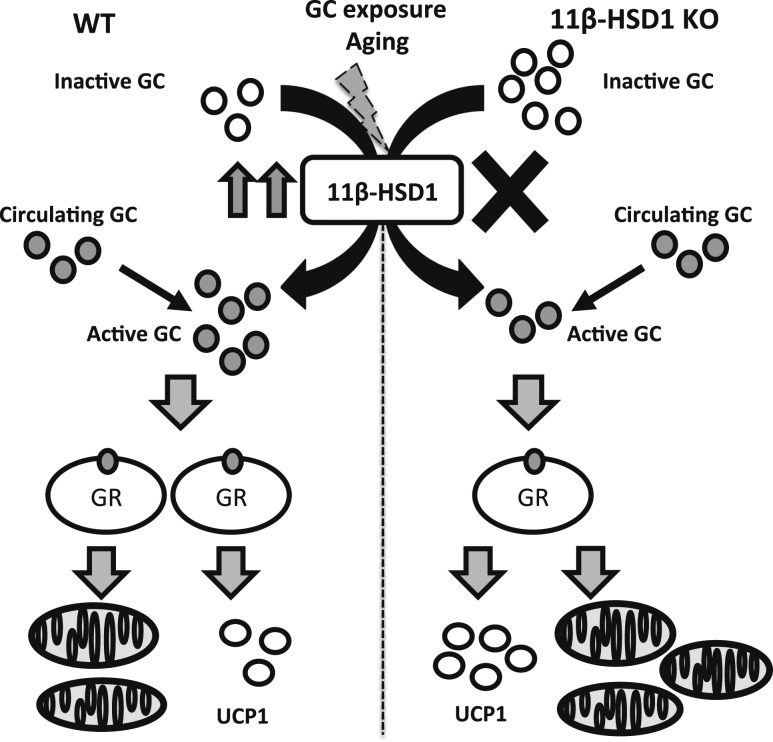
Schematic diagram indicating the impact of excess local GC activation by 11*β*-HSD1 upon BAT function. Stressors such as age related increase in 11*β*-HSD1 expression and chronic excess GC exposure propagates loss of function in mitochondria, mitochondrial biogenesis, and oxidative metabolism of murine BAT.

Exposure of human and mouse BAT to chronically elevated GC levels impairs function by interfering with sympathetic adrenergic signaling and suppression of transcriptional programs that regulate nonshivering thermogenesis (15, 35). Here, we provide evidence that elevated 11*β*-HSD1 expression within brown adipocytes, as a consequence of GC excess and aging, can impair the BAT thermogenic program, mitochondrial biogenesis, and respiratory capacity. As such, 11*β*-HSD1-mediated GC generation can influence the ability of BAT to execute its primary function of thermogenic regulation (Fig. 6).

Within normal physiology, 11*β*-HSD1 performs as a gatekeeper, locally tuning active GC exposure on a tissue-specific basis. Global 11*β*-HSD1 KO mice display cardioprotective phenotypes in the context of high-fat diets or in models of atherosclerosis (36). Little is understood of the *in vivo* role 11*β*-HSD1 plays in BAT physiology other than that 11*β*-HSD1 KO mice have elevated core temperature, and adipose-overexpressing transgenic mice have suppressed UCP1 mRNA expression (25, 37). Tissue-specific GC excess in WT mice, and ensuing metabolic disease (*e.g.*, myopathy, hepatic steatosis, hypertension), is a function of increased circulating delivery of the inactive GC precursor 11-DHC after kidney (and, to a lesser extent, colon and salivary glands) 11*β*-HSD2 activity converting CORT to 11-DHC, and GC-mediated increases in 11*β*-HSD1 expression and activity. However, 11*β*-HSD1 KO mice are protected from this classical metabolic phenotype, underscoring the importance of 11*β*-HSD1 to mediate pathophysiology (28, 38).

We show that both circulating GC excess and advanced age share the common feature of transcriptional upregulation of BAT 11*β*-HSD1 and elevated intracellular GC regeneration, which negatively impact functional markers. The idea that inappropriate 11*β*-HSD1 expression leads to poor BAT function is endorsed in recent findings that the epigenetic regulator lysine-specific demethlyase-1 represses 11*β*-HSD1, such that lysine-specific demethlyase-1 loss of function leads to inappropriately elevated expression and activity to impair BAT mitochondrial metabolic function (33).

UCP1 is critical to the ability of BAT to dissipate chemical energy as heat, and a consistent finding in our data was that 11*β*-HSD1 KO mice, whether young, aged, or when subject to GC excess, displayed elevated UCP1 protein levels, despite mRNA equivalent to WT mice. UCP1 mRNA levels do not always predict the level of protein, and discrepancy between transcriptional output and translational product of UCP1 have been observed previously; thus, here, they may be attributable to a prolonged half-life and extended posttranslational stability (39, 40). Thus, elevated UCP1 protein in 11*β*-HSD1 KO BAT would increase the potential for efficient BAT thermogenic activity, in line with previous work (41).

We provide evidence that a consequence of elevated 11*β*-HSD1 expression and activity in GC excess and aging is the suppression of mitochondrial electron transport chain subunit content, which is rescued in the absence of 11*β*-HSD1. Indeed, 11*β*-HSD KO BAT displays an enhanced transcriptional signature of classical mitochondrial markers and resists suppression of factors influencing mitochondrial biogenesis. GC receptor regulation of nuclear genes involved in mitochondrial energy metabolism has been demonstrated, and mitochondrial localization of GR may also directly influence transcription of mitochondrial genes (42–44). In particular, the identification of various GC-responsive elements contained within the mitochondrial genome has been shown by a number of studies in a variety of tissues (predominantly neuronal, hematopoietic progenitors, and liver cells), including changes in expression of the oxidative phosphorylation complexes (45–47). This leads to the intriguing potential for GC excess within BAT being partially dictated through mtDNA-GR interaction. Evidence exists for a GR-mediated role in mitochondrial-nuclear shuttling, driving differentiation and the adipogenic phenotype (48). As such, these data hint that 11*β*-HSD1 expression may influence mitochondrial function, requiring further investigation.

To assess whether the *in vivo* findings extended to cell autonomous brown adipocyte responses to GC and temperature in the absence of 11*β*-HSD1, we evaluated primary cultures. Again, 11*β*-HSD1 KO cells displayed an enhanced brown adipocyte gene expression profile but were more sensitive to suppression when exogenous GC was administered. *In vivo,* the BAT thermogenic program is stimulated by cold temperatures (2), and cold exposure can rescue metabolic disturbances due to GC excess (18). We show that 11*β*-HSD1 KO-derived brown adipocytes at 30°C had exaggerated transcriptional responses compared with WT adipocytes, highlighting that 11*β*-HSD1 is at least important for establishing basal expression.

11*β*-HSD1KO cells had increased basal and maximal respiratory rates, increased ATP production, and resisted GC-induced suppression. CL316,243 activates *β*-adrenoreceptors in brown adipose (49), and 11*β*-HSD1 KO cells treated with this agonist, while displaying increased absolute basal and maximal oxygen consumption, had similar relative induction to WT-treated cells, implying that lack of 11*β*-HSD1 does not augment *β*-adrenergic signaling in this *in vitro* system. Why cells removed from sympathetic innervation, and circulatory delivery of active and inactive GC, display a cell autonomous phenotype is not entirely clear. The primary cultures were generated in the presence of fetal bovine serum (refreshed daily), which contained GCs (50). Additionally, the cocktail of factors supporting primary brown adipocytes contains dexamethasone (a prerequisite for differentiation), which, in WT cells, could maximize 11*β*-HSD1 expression and activity and further enhance GC activation, with 11*β*-HSD1KO cells protected from these effects.

Concomitant with aging, 11*β*-HSD1 expression is elevated in numerous tissues, such as skin and brain regions, and we demonstrate this to be true for BAT (51, 52). A mechanism for increased 11*β*-HSD1 expression with age is not clear and may reflect tissue-specific responses to inflammatory signals, alterations in the epigenetic landscape, hormone decline, chronic stress, and alterations in HPA axis activity that subtly but chronically increase GC exposure (30). With this in mind, we present evidence that aged BAT from 11*β*-HSD1 KO mice displays a profile in keeping with greater thermogenic potential than age-matched WT mice. UCP1 protein was increased, as was relative mitochondrial copy number and electron transport chain protein content. Furthermore, aged tissue appears to display greater levels of phosphorylated CREB, which may imply enhanced thermogenic potential acting through the *β*-adrenoreceptor pathway (53). GCs are known to interfere with this signaling pathway (54, 55). Although we did not observe this protection in the context of GC excess, lifelong 11*β*-HSD1 deficiency may alter tone sufficiently to increase CREB phosphorylation.

The emerging species differences between humans and mice in terms of acute UCP1 and BAT thermogenic responses to GC is intriguing and may yet yield important insights to human BAT physiology and have implications for future translational work (5, 6). In addition, acute dexamethasone enhances 11*β*-HSD1 gene expression in human BAT to a greater extent than in white adipose tissue, which also poses further implications when delineating results between species (6). However, chronic GC exposure appears to have the same impact on BAT for both species, endorsing the utility of the data presented here.

In summary, our data support the idea that 11*β*-HSD1 can set the sensitivity of murine BAT to GC, and that elevated 11*β*-HSD1 expression contributes to the pathophysiological mechanisms negatively impacting BAT thermogenic capacity in the context of GC excess and aging.

## Appendix

**Appendix.** **Antibody Table**

**Peptide/Protein Target**	**Antibody**	**Manufacturer, Catalog No.; RRID**	**Species Raised in**	**Dilution**
UCP1	UCP1	Abcam, Ab23841; RRID 2213764	Rabbit	1/10,000
Mitochondrial OXPHOS subunits (1-5)	OXPHOS cocktail	Abcam, Ab110413; RRID AB_2629281	Rodent	1/10,000
*β*-actin	*β*-actin	Abcam, Ab8226; RRID AB_306371	Mouse	1/10,000
CREB total	CREB total	Cell Signaling, 9197; RRID AB_331277	Rabbit	1/1000
Phos-CREB Ser-133	Phos-CREB Ser-133	Cell Signaling, 9198; RRID AB 256044	Rabbit	1/1000
11*β*-HSD1	11*β*-HSD1	Abcam, Ab39364; RRID AB_731458	Rabbit	1/1000
