# Supplementary figures and images for: 11β-HSD1 Modulates the Set Point of Brown Adipose Tissue Response to Glucocorticoids in Male Mice
Source: Endocrinology. 2017 Mar 27;158(6):1964–76. doi: 10.1210/en.2016-1722 (PMC5460930; doi:10.1210/en.2016-1722)

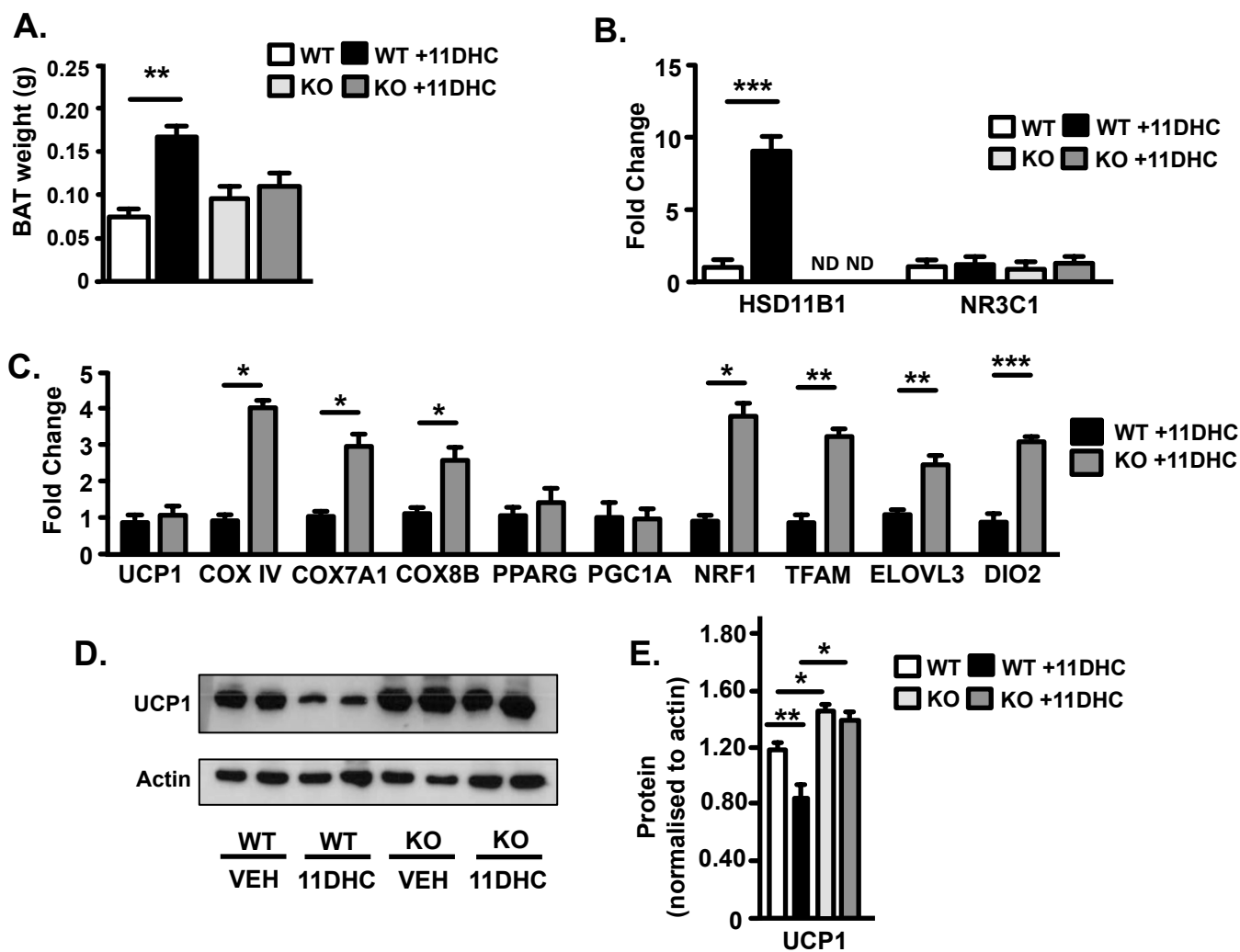

**Supplementary Figure 1.**

Supplement: Supplementary file 1 [file en.2016-1722.sf1.pdf]
